# Prognostic significance and value of further classification of lymphovascular invasion in invasive breast cancer: a retrospective observational study

**DOI:** 10.1007/s10549-024-07318-6

**Published:** 2024-05-21

**Authors:** Yuyang Zhang, Huali Wang, Huahui Zhao, Xueming He, Ya Wang, Hongjiang Wang

**Affiliations:** 1 Department of Breast Surgery, The First Affiliated Hospital of Dalian Medical University, No. 193, Union Road, Shahekou District, Dalian, Liaoning China; 2 Department of Pathology, The First Affiliated Hospital of Dalian Medical University, Dalian, China

**Keywords:** Breast cancer, Lymphovascular invasion, Prognosis, Specific vascular endothelial marker

## Abstract

**Purpose:**

To investigate the prognostic significance of lymphovascular invasion in invasive breast cancer and the value of using specific vascular endothelial markers to further classify lymphovascular invasion.

**Methods:**

We collected 2124 patients with invasive breast cancer who were hospitalized at the First Hospital of Dalian Medical University from 2012 to 2020. Statistical methods were used to investigate the relationship between lymphovascular invasion and clinicopathological characteristics of breast cancer, and the correlation between lymphovascular invasion on overall survival (OS) and disease-free survival (DFS) of various categories of breast cancers. Immunohistochemical staining of breast cancer samples containing lymphovascular invasion using specific vascular endothelial markers D2-40 and CD34 was used to classify lymphovascular invasion and to investigate the relationship between lymphovascular invasion and breast cancer progression.

**Results:**

There was a high correlation between lymphovascular invasion and T stage, N stage and nerve invasion. Survival analyses showed that patients with lymphovascular invasion, especially luminal B, triple-negative, and Her-2 overexpression breast cancer patients, had poorer OS and DFS prognosis, and that lymphovascular invasion was an independent prognostic factor affecting OS and DFS in breast cancer. The immunohistochemical staining results showed that positive D2-40 staining of lymphovascular invasion was linked to the N stage and localized recurrence of breast cancer.

**Conclusion:**

Lymphovascular invasion is associated with aggressive clinicopathological features and is an independent poor prognostic factor in invasive breast cancer. Breast cancer localized recurrence rate and lymph node metastases are influenced by lymphatic vessel invasion. Immunohistochemical techniques should be added to the routine diagnosis of lymphovascular invasion.

## Introduction

Based on the worldwide cancer data released by CA Cancer J Clin 2021[1], breast cancer is the most prevalent malignant tumor worldwide at the moment. According to the data, breast cancer accounts for 15.5 percent of cancer mortality in women, which is higher than other types of cancers such as lung, colorectal, and cervical cancer. The diagnosis and treatment of breast cancer have evolved as we approach the era of precision therapy. An exact disease risk assessment is essential to creating a tailored breast cancer therapy regimen. However, conventional predictive risk markers like TNM staging and tumor histological grading have not demonstrated high clinical utility in cases of early-stage breast cancer. Therefore, they are no longer the sole criteria for prognostic assessment and therapeutic targets for breast cancer [2].

Lymphovascular invasion (LVI), an important component of the tumor microenvironment, has previously been limited by the limitations of detection techniques. The most common method for testing for LVI in prior research has been hematoxylin–eosin (H&E) staining. However, as immunohistochemistry (IHC) technology has advanced, the objective detection rate of LVI in breast cancer has been further enhanced by the combination of IHC detection with H&E staining [3], which has advanced LVI research. There is disagreement concerning the findings of earlier research on LVI. Lee et al. [4] concluded that LVI is an independent factor for poor prognosis in patients with early-stage breast cancer, regardless of lymph node status and molecular subtype. A study by Houvenaeghel et al. [5] showed that the presence of LVI had an independent negative impact on the prognosis of patients with early-stage breast cancer, except for ER-positive grade 3 tumors and luminal A tumors treated with adjuvant chemotherapy. However, Bent et al. [6] concluded that LVI could not be used as an independent risk factor for breast cancer and was insufficient to move patients from a low to a high risk of recurrence. Munzone et al. [7] discovered that in patients with lymph node-positive breast cancer, LVI had no effect on the overall prognosis but did increase the probability of localized recurrence. Meanwhile, other researchers have focused their research on the influence of LVI on the selection of breast cancer therapy strategies. Zhong et al. [8] demonstrated that patients with early-stage LVI positive breast cancer who undergo breast-conserving surgery have a worse prognosis than patients with LVI negative breast cancer. They concluded that these patients should receive extensive systemic therapy in addition to a mastectomy as part of their treatment. The American Joint Committee on Cancer (AJCC), a reputable prognostic rating method, does not include LVI based on the outcomes of currently available research. Consequently, additional research is still required to confirm the predictive significance of LVI for breast cancer.

Pathologists have employed endothelial cells with various molecular characteristics in recent years to further objectively and standardly define LVI using IHC technology and specific vascular endothelial markers. The growing recognition of the significance of lymphatic endothelial cells in tumor progression and metastasis has been made possible by the development of markers for these cells [9]. On the other hand, since the principle of anti-angiogenic therapy for solid tumors was proposed in 1972 [10], targeted anti-angiogenic drugs combined with conventional chemotherapy and radiotherapy have positively influenced the treatment of tumors as an anti-tumor therapeutic strategy and great breakthroughs have been made in the study of breast cancer-related angiogenesis. Many vascular endothelial markers specific to breast cancer have emerged, and some of these markers have been gradually employed to help diagnose breast cancer-related LVI, improving upon the limitations of conventional H&E staining in this regard. Therefore, researching cancer-related lymphatic and blood vessels using endothelial markers and IHC technology can help to further explore the role that LVI plays in the progression of breast cancer.

In this study, to determine if LVI can be utilized in addition to conventional clinicopathological criteria to help determine the prognosis of breast cancer, we analyzed the association between LVI and clinicopathological characteristics and prognosis in invasive breast cancer. Simultaneously, we labeled LVI in breast cancer using specific vascular endothelial markers to investigate the mechanism of LVI development. The objectives of our research were to investigate the predictive significance of LVI in breast cancer and to offer a theoretical framework for the development of appropriate therapeutic options.

## Materials and methods

### Data collection and processing

We collected a total of 2479 invasive breast cancer cases hospitalized in the Department of Breast Surgery of the First Hospital of Dalian Medical University from January 2012 to December 2020. These patients were subsequently screened using the following inclusion and exclusion criteria. Inclusion criteria: (1) patients who were hospitalized for the first time for radical breast cancer surgery with postoperative pathological confirmation of invasive breast cancer; (2) complete clinicopathological and follow-up data. Exclusion criteria: (1) patients with adjuvant treatment such as neoadjuvant chemotherapy before surgery; (2) special types of breast cancer such as inflammatory breast cancer and Paget's disease of the breast; (3) Stage IV breast cancer; (4) bilateral invasive breast cancer; (5) patients who could not undergo radical mastectomy for breast cancer due to poorer physical status, etc.; (6) presence of a history of breast tumor or other types of tumors; and (7) missing clinicopathological and follow-up data. Finally, 2124 eligible cases were screened, of which 397 cases showed the presence of LVI by H&E staining. The clinicopathological characteristics of the enrolled cases are shown in Table 1.

**Table 1:** Baseline information table for breast cancer patients

Characteristics	Levels	Amount(n = 2124)	Percentage(%)
LVI	Positive	397	18.7
	Negative	1727	81.3
Age	< 40	194	9.1
	≥ 40	1930	90.9
Anatomic neoplasm subdivisions	Left	1099	51.7
	Right	1025	48.3
Menopause status	Premenopausal	800	37.7
	Postmenopausal	1291	60.8
	Hysterectomy	33	1.5
Histological type	Infiltrating ductal carcinoma	1969	92.7
	Infiltrating lobular carcinoma	60	2.8
	Other types	95	4.5
Histological grade	I	49	2.3
	II	1030	48.5
	III	917	43.2
	Not available	128	6.0
T stage	T1	1182	55.6
	T2	858	40.4
	T3	61	2.9
	T4	23	1.1
N stage	N0	1253	59.0
	N1	560	26.4
	N2	176	8.3
	N3	135	6.3
PAM50	Luminal A	404	19.0
	Luminal B	1289	60.7
	Triple-negative	261	12.3
	Her-2 overexpressing	170	8.0
ER status	Negative	471	22.2
	Weakly positive	74	3.5
	Positive	1579	74.3
PR status	Negative	593	27.9
	Weakly positive	186	8.8
	Positive	1345	63.3
Ki67 status	≤ 14%	496	23.4
	> 14%	1628	76.6
HER-2 status	Negative	1673	78.8
	Positive	451	21.2
Pathologic stage	Stage I	841	39.6
	Stage II	948	44.6
	Stage III	335	15.8
Neurological invasion	Positive	212	10.0
	Negative	1912	90.0
Soft tissue invasion	Positive	73	3.4
	Negative	2051	96.6

### Assessment of LVI

According to the Chinese guidelines for the diagnosis and treatment of breast cancer, the presence or absence of LVI in the lesions of breast cancer patients should be routinely included in the postoperative pathology reports after they have undergone the appropriate radical mastectomy for breast cancer. The final diagnosis of LVI in all the cases in this study was based on the official pathology reports published by the Department of Pathology of the First Affiliated Hospital of Dalian Medical University. The pathological evaluation of LVI in breast cancer is as follows: using H&E staining, LVI is diagnosed if there are clusters of tumor cells in the lymphovascular lumen (including lymphatic lumen or vascular lumen) within 1 mm around the tumor, and it should be noted that due to the fixation of tissues, H&E staining is unable to differentiate between LVI and contractile space. Therefore, in the few cases where it is not possible to confirm whether the lumen surrounding the tumor is a lymphovascular lumen, additional immunohistochemical staining should be performed to assist in the diagnosis of this indistinguishable pathological tissue.

### Pathological sample collection

245 paraffin-embedded breast cancer tissues from January 2016 to December 2020 were collected by the Department of Pathology of the First Hospital of Dalian Medical University. Under H&E staining, these tissues previously showed the existence of LVI.

### Immunohistochemistry

Immunohistochemical staining using the Envision [11] technique as a guide. The following are the precise steps: Breast cancer tissues were fixed in 10% formalin, paraffin-embedded, and sectioned to 4-6 μm on slides. After dewaxing, hydration, and microwave antigen repair of the slides, an endogenous peroxidase blocker (3% hydrogen peroxide) was added. Slides were incubated overnight at 4 °C with the D2-40/CD34 antibody. Subsequently, the slides were incubated with the secondary antibody for 20 min at room temperature. DAB chromogenic stain, then hematoxylin re-staining, hydrochloric acid alcohol differentiation, and return to blue. Finally, the sections were dehydrated, transparent, and sealed. The stained sections were observed and the results were interpreted under a light microscope. The discriminatory criterion for D2-40/CD34 positivity is to specify the presence of positive staining (brown-yellow staining) of vascular endothelial cells. Under the microscope, vascular endothelial cells in the lumen of a small vessel (lymphatic lumen or vascular lumen) surrounding the LVI tissue are positive for D2-40/CD34 if they show brown-yellow staining, and negative for D2-40/CD34 if they do not show brown-yellow staining. All the experimental reagents involved in the immunohistochemical staining process were purchased from ZSGB-BIO.

### Follow-up visit

Two follow-up visits were conducted in this study, and all cases were followed up by telephone or outpatient review. The first follow-up: November 2021. Follow-up target: all cases meeting the enrollment criteria. Second follow-up: November 2023. Follow-up target: 245 breast cancer patients corresponding to the collected pathological samples. In this study, overall survival (OS) and disease-free survival (DFS) were used as indicators to assess the prognosis of breast cancer patients.

### Survival analysis

Survival analyses were performed using the Kaplan–Meier method and log-rank test, with the cut-off value set in the presence of LVI. Univariate and multivariate Cox regression analyses were used to assess the effect of clinical variables on patients' OS and DFS. Prognostic variables with *P* < 0.05 in the univariate Cox regression analysis were included in the multivariate Cox regression analysis. Forest plots were visualized using the R package ggplot2 [12].

### Statistical analysis

The xiantao platform (https://www.xiantao.love), built on R, was used to perform the statistical analyses. All statistical analyses were performed using R (version 4.2.1). The correlation between LVI and clinicopathological factors was analyzed using the chi-square test, and factors satisfying *P* < 0.05 in the chi-square test were included in the multivariate logistic regression. The correlations between D2-40 staining of LVI and N stage, localized recurrence of breast cancer, as well as the correlation between CD34 staining and organ metastasis of breast cancer, were examined using the chi-square test. All tests were two-sided, and *P* < 0.05 was considered statistically significant.

## Results

### Correlation between LVI and clinicopathological factors

**Fig. 1:**
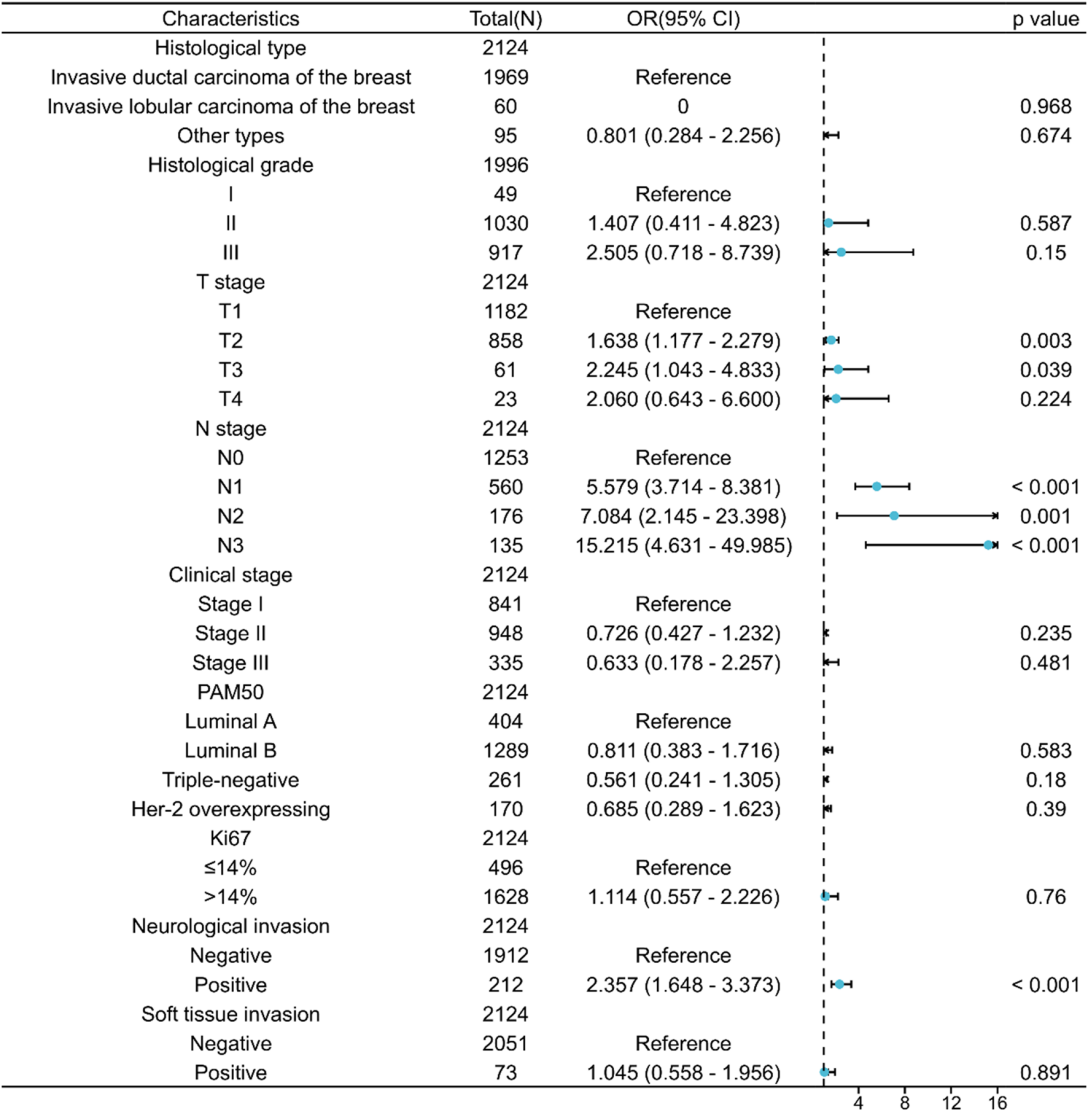
Forest map based on logistic models associated with LVI

Univariate analysis showed that LVI in breast cancer was associated with T stage, N stage, histological type, histological grade, pathological molecular type, Ki-67 status, pathologic stage, neurological invasion, and soft tissue invasion (*P* < 0.05). LVI was not associated with anatomic neoplasm subdivisions, menopause status, age, ER status, PR status, and Her-2 status (Table 2). A multivariate logistic regression model and forest map based on the association of LVI showed that LVI was strongly associated with T stage, N stage, and neurological invasion (*P* < 0.05) (Fig. 1).

**Table 2:** Clinicopathological characteristics of LVI positive and LVI negative expression groups

Characteristics	Levels	LVI positive	LVI negative	*P* value
Anatomic neoplasm subdivisions, n (%)	Right	205(9.7%)	820(38.6%)	0.135
	Left	192(9%)	907(42.7%)	
Age, n (%)	≥ 40	357(16.8%)	1573(74.1%)	0.470
	< 40	40(1.9%)	154(7.3%)	
Menopause status, n (%)	Postmenopausal	244(11.5%)	1047(49.3%)	0.849
	Premenopausal	148(7%)	652(30.7%)	
	Hysterectomy	5(0.2%)	28(1.3%)	
Histological type, n (%)	Infiltrating ductal carcinoma	377(17.7%)	1592(75%)	0.003
	Infiltrating lobular carcinoma	1(0%)	59(2.8%)	
	Other types	19(0.9%)	76(3.6%)	
Histological grade, n (%)	I	3(0.2%)	46(2.3%)	< 0.001
	II	153(7.7%)	877(43.9%)	
	III	223(11.2%)	694(34.8%)	
T stage, n (%)	T1	148(7%)	1034(48.7%)	< 0.001
	T2	212(10%)	646(30.4%)	
	T3	26(1.2%)	35(1.6%)	
	T4	11(0.5%)	12(0.6%)	
N stage, n (%)	N0	92(4.3%)	1161(54.7%)	< 0.001
	N1	167(7.9%)	393(18.5%)	
	N2	62(2.9%)	114(5.4%)	
	N3	76(3.6%)	59(2.8%)	
Pathologic stage, n (%)	Stage I	60(2.8%)	781(36.8%)	< 0.001
	Stage II	189(8.9%)	759(35.7%)	
	Stage III	148(7%)	187(8.8%)	
PAM50, n (%)	Luminal A	52(2.4%)	352(16.6%)	0.005
	Luminal B	263(12.4%)	1026(48.3%)	
	Triple-negative	45(2.1%)	216(10.2%)	
	Her-2 overexpressing	37(1.7%)	133(6.3%)	
ER status, n (%)	Positive	288(13.6%)	1291(60.8%)	0.442
	Negative	97(4.6%)	374(17.6%)	
	Weakly positive	12(0.6%)	62(2.9%)	
PR status, n (%)	Positive	246(11.6%)	1099(51.7%)	0.823
	Negative	115(5.4%)	478(22.5%)	
	Weakly positive	36(1.7%)	150(7.1%)	
Ki67 status, n (%)	≤ 14%	66(3.1%)	430(20.2%)	< 0.001
	> 14%	331(15.6%)	1297(61.1%)	
HER-2 status, n (%)	Positive	98(4.6%)	353(16.6%)	0.062
	Negative	299(14.1%)	1374(64.7%)	
Neurological invasion, n (%)	Positive	78(3.7%)	134(6.3%)	< 0.001
	Negative	319(15%)	1593(75%)	
Soft tissue invasion, n (%)	Positive	30(1.4%)	43(2%)	< 0.001
	Negative	367(17.3%)	1684(79.3%)

### Prognostic value of LVI in breast cancer

**Fig. 2:**
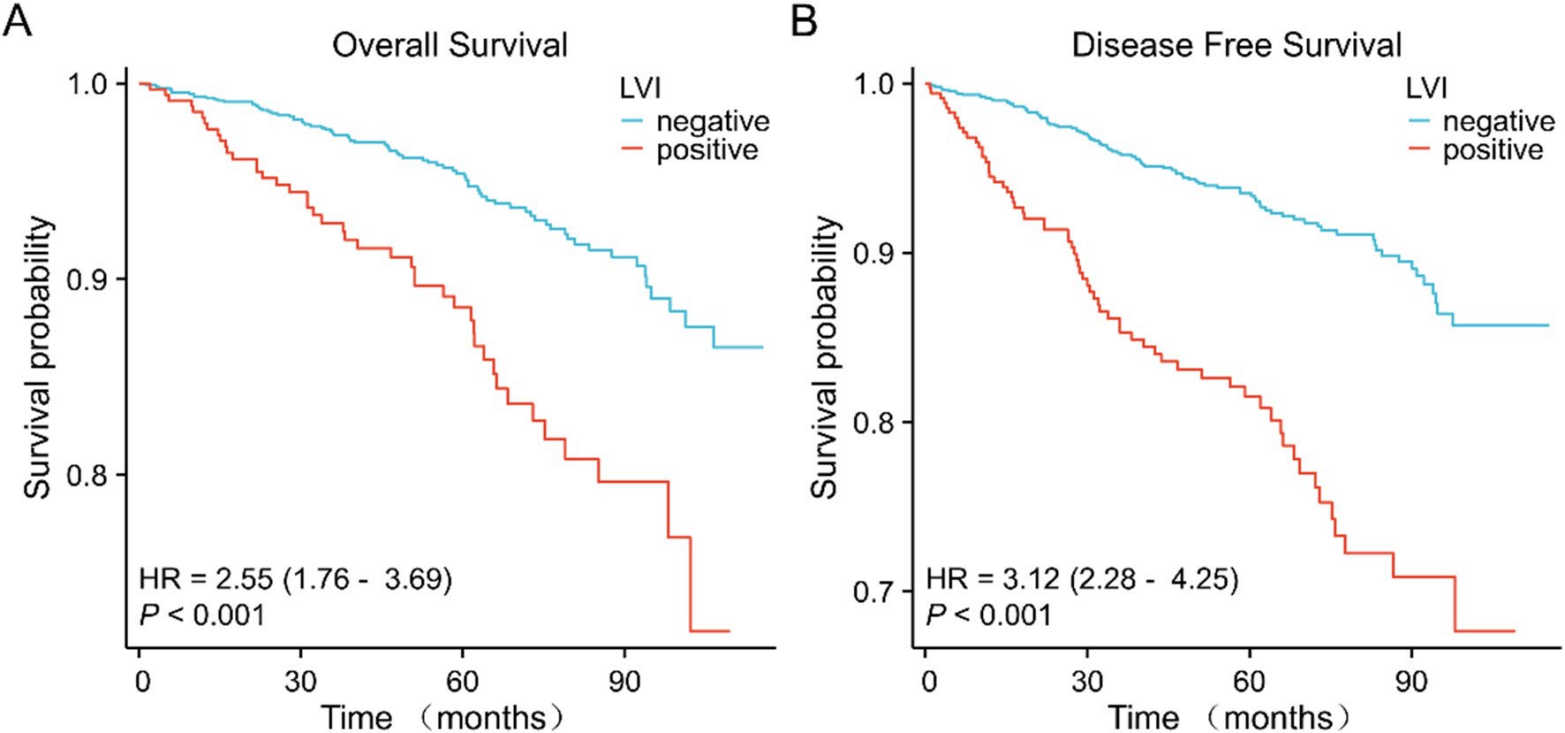
Prognostic values of LVI in patients with breast cancer evaluated by the Kaplan–Meier method. Overall survival (**A**) and disease-specific survival (**B**) for breast cancer patients with or without LVI

**Fig. 3:**
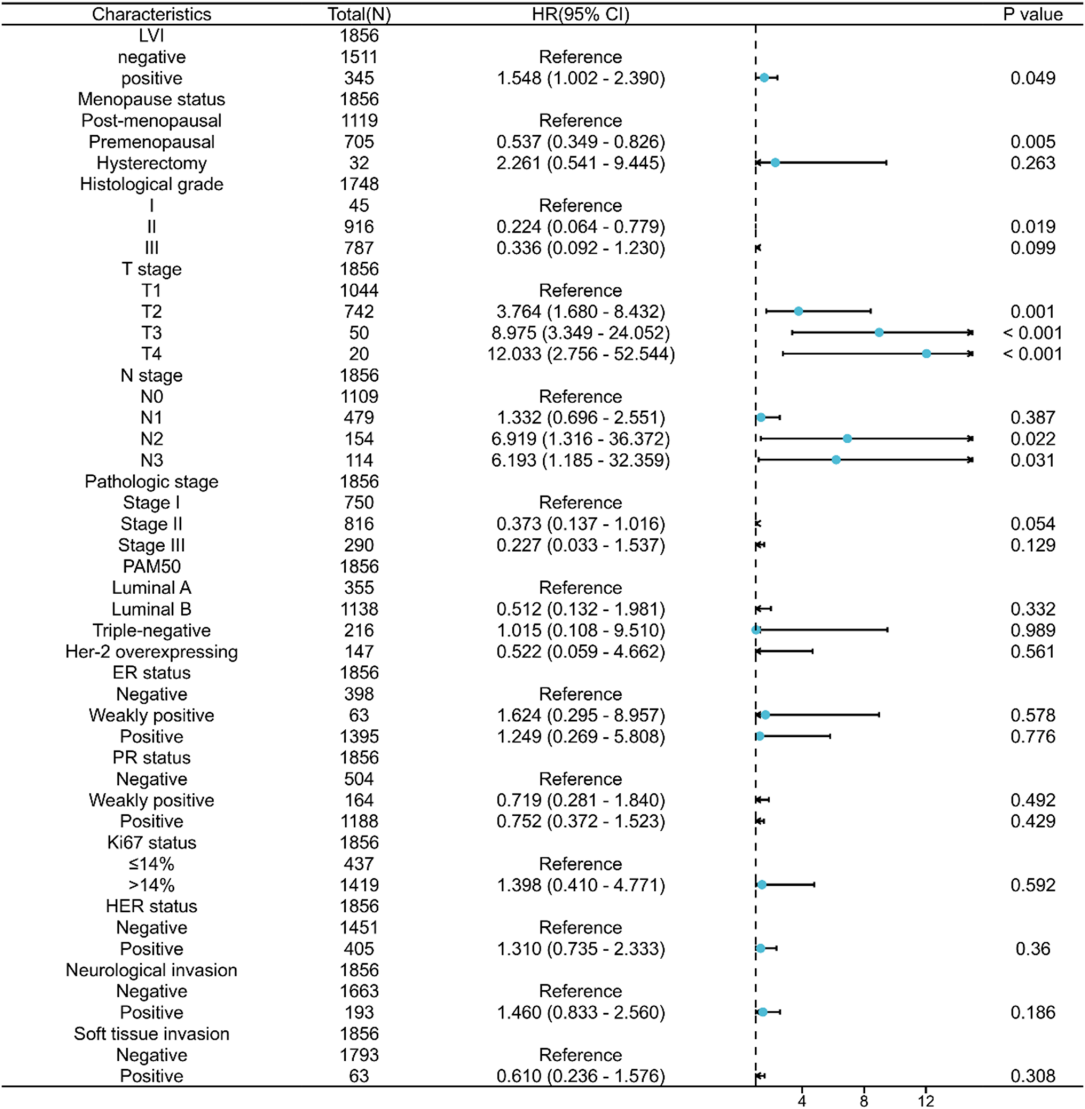
Forest map based on multivariate Cox analysis for overall survival

**Fig. 4:**
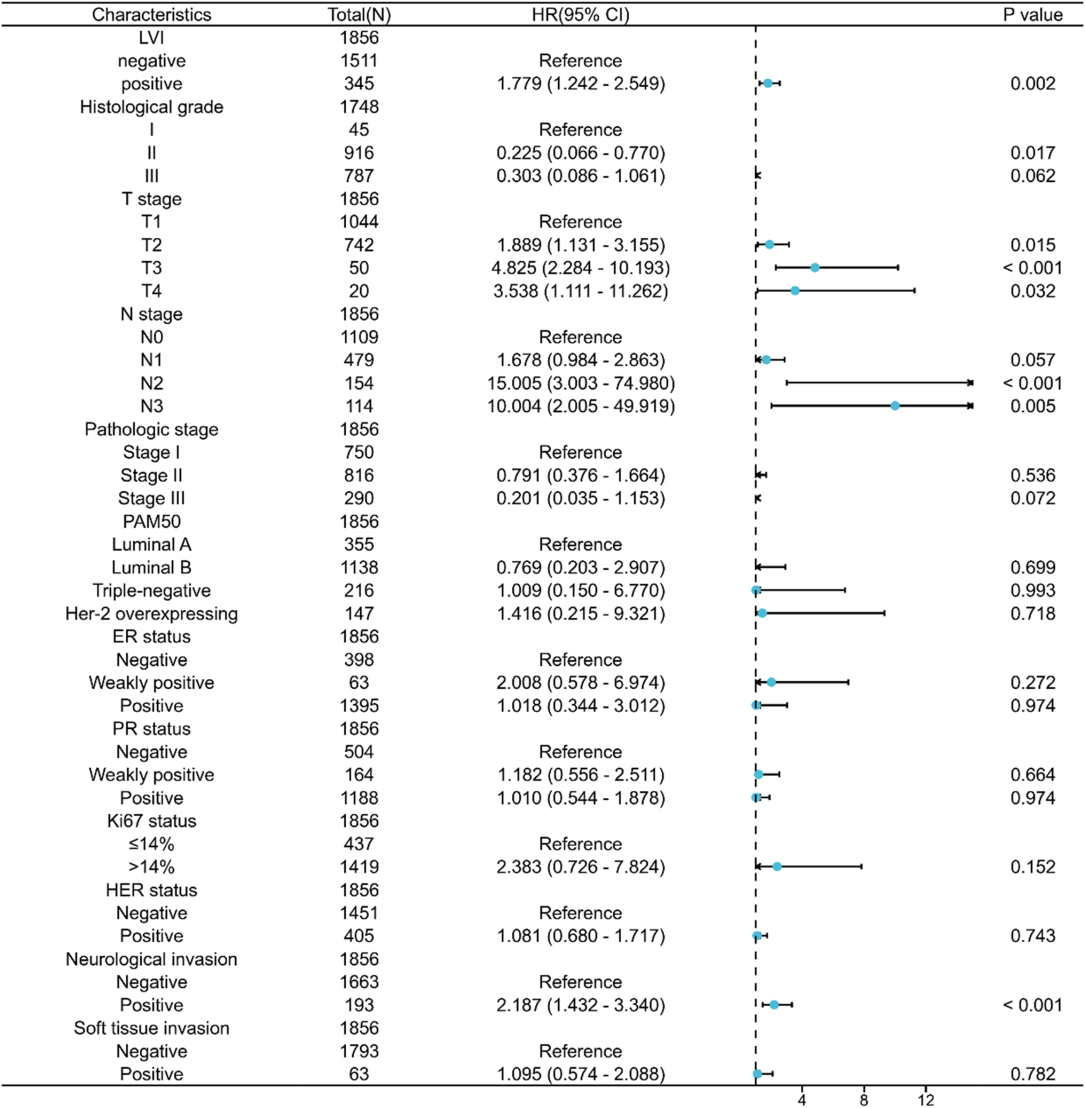
Forest map based on multivariate Cox analysis for disease-free survival

Survival analyses showed that patients with LVI had a worse prognosis for both OS and DFS compared with patients without LVI (OS: hazard ratio [HR] = 2.55, 95%CI = 1.76–3.69, *P* < 0.001; DFS: HR = 3.12, 95% CI = 2.28–4.25, *P* < 0.001) (Fig. 2A, B). The results of multivariate Cox regression analysis showed that LVI was an independent prognostic factor affecting OS and DFS in invasive breast cancer (OS: adjusted HR = 1.548, 95% CI = 1.002–2.390, *P* = 0.049; DFS: adjusted HR = 1.779, 95% CI = 1.242–2.549, *P* = 0.002) (Fig. 3, Fig. 4).

### Prognostic value of LVI in different subgroups of breast cancer

**Fig. 5:**
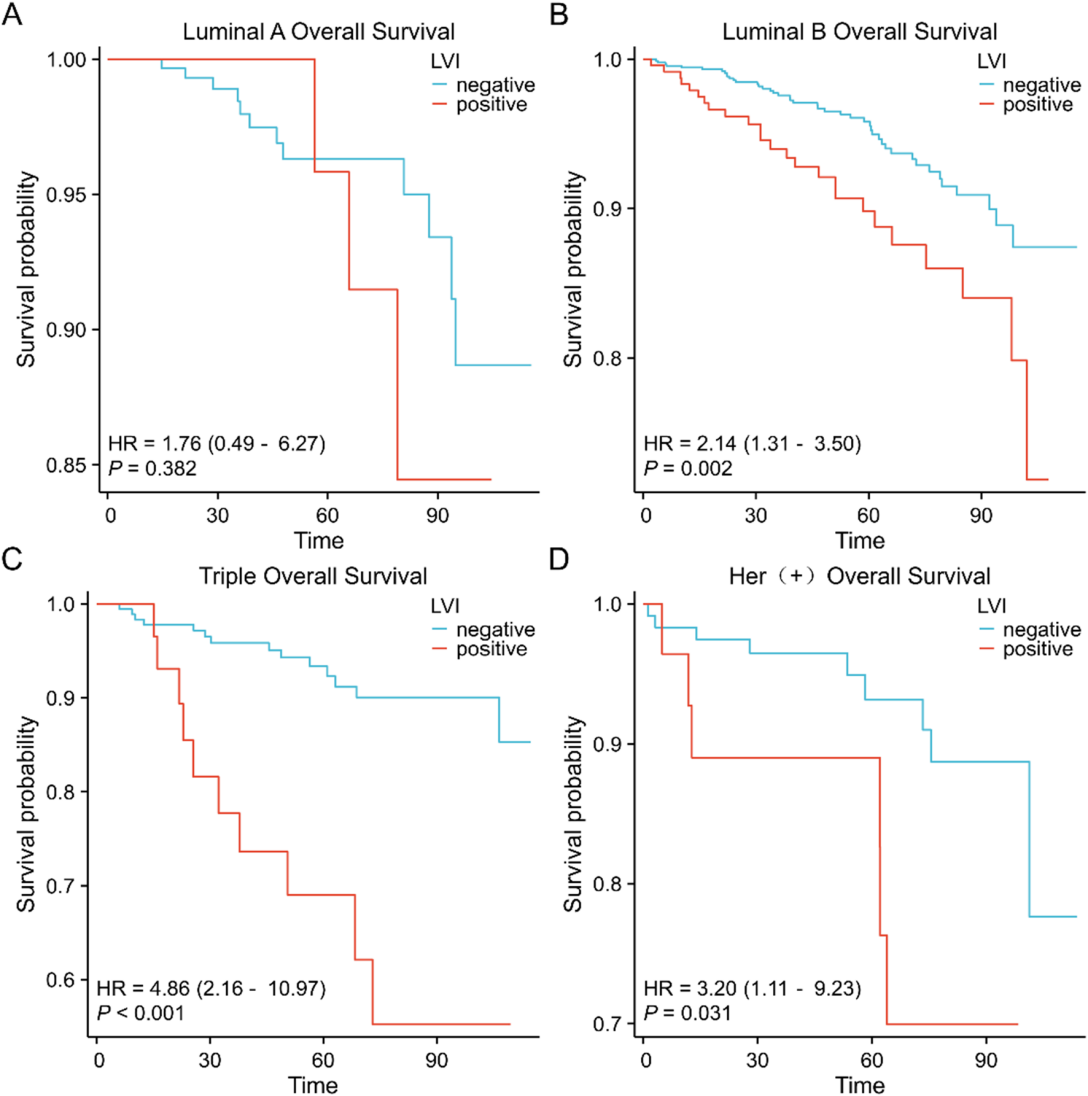
Prognostic value of LVI on overall survival in different subgroups of breast cancer evaluated by the Kaplan–Meier method **A**. luminal A breast cancer **B**. luminal B breast cancer **C**. triple-negative breast cancer **D**. Her-2 overexpressing breast cancer

**Fig. 6:**
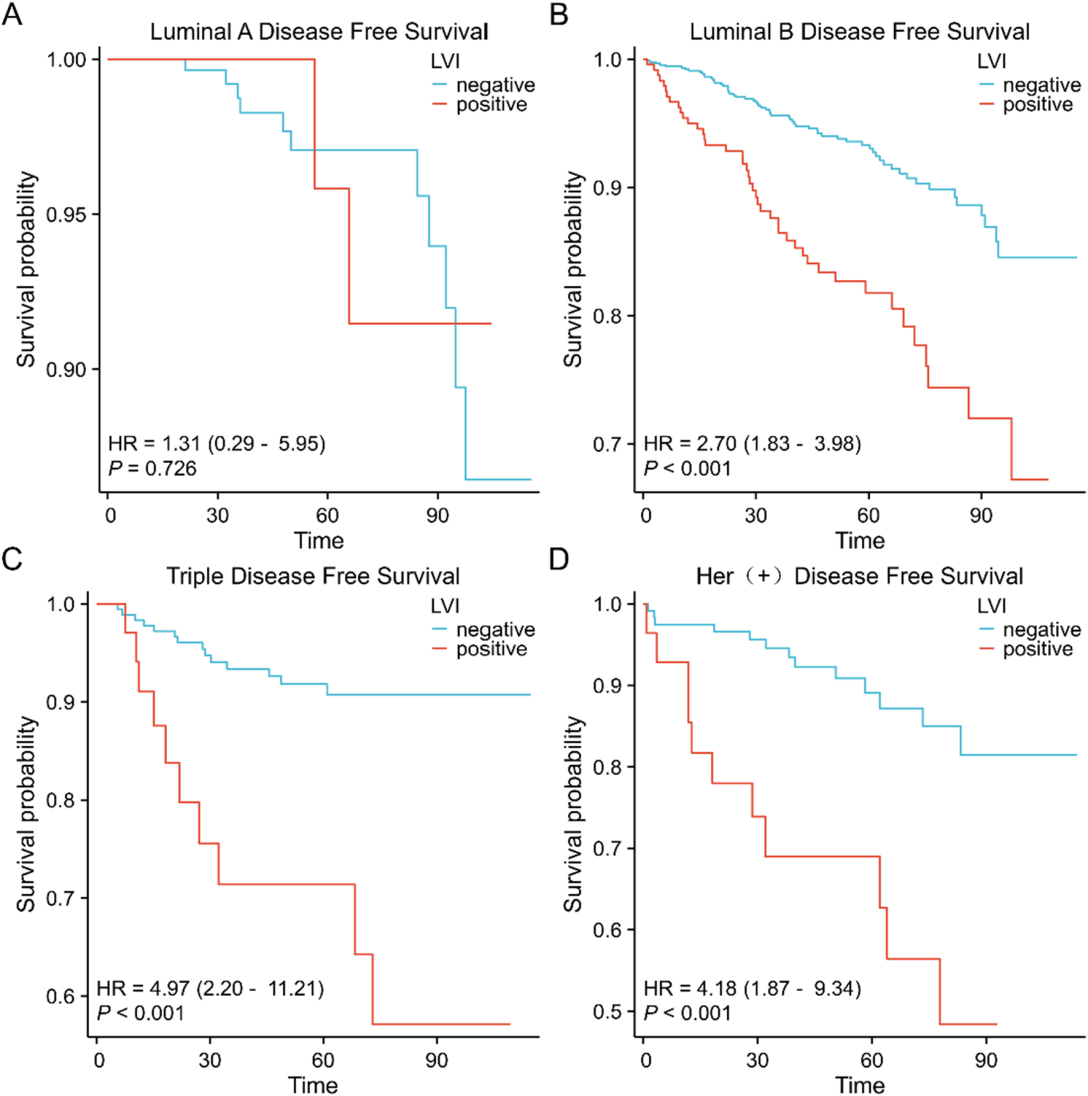
Prognostic value of LVI on disease-free survival in different subgroups of breast cancer evaluated by the Kaplan–Meier method **A**. luminal A breast cancer **B**. luminal B breast cancer **C**. triple-negative breast cancer **D**. Her-2 overexpressing breast cancer

Survival analyses in different subgroups of breast cancer showed that patients with LVI had a worse OS and DFS prognosis in luminal B, triple-negative, and Her-2 overexpressing breast cancers. In luminal A breast cancer, the difference in OS and DFS prognosis between patients with LVI and controls was not statistically significant (Figs. 5A–D, 6A–D).

### The value of specific vascular endothelial markers

**Fig. 7:**
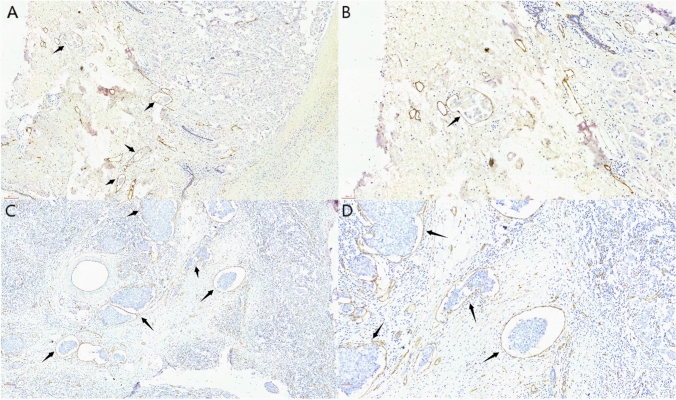
Immunohistochemical staining of D2-40 and CD34 of LVI in breast cancer (arrowed) **A**. D2-40 staining of LVI at 10× **B**. D2-40 staining of LVI at 20× **C**. CD34 staining of LVI at 10× **D**. CD34 staining of LVI at 20x

We used D2-40 versus CD34 for immunohistochemical labeling of each of the 245 paraffin-embedded breast cancer tissues. Of these tissues, 68 had positive staining for D2-40, while 50 had positive staining for CD34. Representative images are shown in Fig. 7 (Fig. 7A–D). Chi-square analysis results indicated that positive D2-40 staining of LVI was linked to both N stage and localized recurrence of breast cancer. On the other hand, there was no correlation found between positive CD34 staining of LVI and organ metastases of breast cancer (Table 3, 4).

**Table 3:** Correlations of D2-40 staining of LVI with N stage and localized recurrence of breast cancer

Characteristics	Negative expression of D2-40	Positive expression of D2-40	*P* value
N stage, n (%)			0.013
N0	45 (18.4%)	11 (4.5%)	
N1	79 (32.2%)	28 (11.4%)	
N2	31 (12.7%)	9 (3.7%)	
N3	22 (9%)	20 (8.2%)	
Localized recurrence, n (%)			0.034
Normal	166 (67.8%)	58 (23.7%)	
Recurrence	11 (4.5%)	10 (4.1%)

**Table 4:** Correlation of CD34 staining of LVI with organ metastasis of breast cancer

Characteristics	Negative expression of CD34	Positive expression of CD34	*P* value
Tumor organ metastases, n (%)			0.234
Normal	167 (68.2%)	46 (18.8%)	
Metastasis	28 (11.4%)	4 (1.6%)	

## Discussion

Clinicopathological characteristics that predict the prognostic risk of cancer are responsible for the improved survival results that breast cancer patients experience under the present therapy paradigm. Through effective targeting, clinicians are provided with appropriate therapeutic approaches for patients with breast cancer in various risk groups. Precision medicine is now the standard of care for breast cancer patients, and creating effective treatment regimens requires accurate risk classification of the illness. Conventional prognostic risk indicators, like TNM staging and histological grading, are well known and used in reputable prognostic evaluation systems for breast cancer, like the AJCC [13]. However, these factors did not show high prognostic value in specific categories of breast cancer. This suggests that current staging systems are still insufficient to accurately predict prognosis and accurately reflect the biological heterogeneity of breast cancer [14, 15].

The essential condition for the systematic spread of breast cancer cells to other regions is that they must first penetrate and spread throughout the lymphovascular system [16]. Tumor cells in the endothelial lining space of lymphatic vessels or blood vessels surrounding the primary tumor might enter adjacent lymphatic vessels or blood vessels and bind to one another, forming a tumor embolism known as LVI [17]. The College of American Pathologists recommends assessing and reporting LVI in all cancer protocols, which is the gold standard for cancer reporting [18]. Though its predictive utility in breast cancer is still debatable, LVI certainly plays a role in treatment selection for breast cancer. For patients with LVI, the St. Gallen International Consensus Guidelines recommend whole-breast irradiation as opposed to localized irradiation [19]. According to recent investigations, the 21-gene recurrence score can be reliably determined by detecting LVI [20]. Consequently, knowing how LVI affects breast cancer patient survival and the progression of the disease may lead to new therapeutic and diagnostic approaches that will enhance the prognosis of breast cancer patients.

In this study, we collected data from approximately two thousand invasive breast cancer patients who had therapy at our medical center during the previous nine years. Consistent with earlier studies [21], statistical analysis revealed that LVI was linked to detrimental clinicopathological characteristics, such as T stage, N stage, and neurological invasion. Tumor size and lymph node metastasis of breast cancer are intuitive bases for predicting cancer recurrence and metastasis, both of which are closely related to the degree of differentiation of the tumor cells and the invasive ability of the cancer. Our study's findings indicate that even in the absence of lymph node metastases, breast cancer patients with LVI are at risk for recurrence, so it's important to evaluate carefully whether postoperative adjuvant radiation therapy should be used in these cases following the recommended course of treatment for lymph node metastases.

Key indicators for evaluating the prognosis of patients are the overall and disease-free survival rates for breast cancer. In our study, breast cancer patients with LVI had worse OS and DFS prognosis. LVI predicts a poor prognosis in all subtypes of breast cancer, except luminal A. LVI, T stage, N stage, and menstrual status were independent predictive biomarkers of poor OS and DFS in patients with invasive breast cancer, according to survival analysis. Our research indicates that, when it comes to determining the predictive risk of breast cancer, LVI is not less useful than conventional clinicopathological factors such as tumor size and lymph node metastatic status. As a result, when formulating treatment plans, physicians shouldn't ignore the prognostic significance of LVI in breast cancer. In the meanwhile, patients having LVI for breast cancer may have an increased chance of developing recurring metastases. These individuals ought to be categorized as having a medium or high risk of developing new cancer metastases, and they ought to be the target of appropriate adjuvant therapy plans.

Malignant tumor cells extrude the junction of lymphatic endothelial cells (LEC) and move along the LEC to the subsequent stop lymph nodes and organs during lymphatic metastasis of breast cancer [9]. Tumor cells use LEC to facilitate malignancy invasion within the tumor microenvironment [22]. Kahn et al. [23] identified the monoclonal antibody D2-40 as a lymphatic vessel endothelial marker in 2002. When it comes to assessing lymphatic vessel invasion, D2-40 outperforms H&E staining in two ways: first, it can identify tumor emboli that are obstructing the lymphatic vessel lumen; second, it can distinguish between tumor aggregates because of tissue contraction during fixation and retraction artifacts that separate tumor emboli in the lymphatic vessel space. D2-40 enhanced the detection of lymphatic vessel invasion in primary tumors, according to Kahn's study. Debald et al. [24] recommended routine D2-40 immunohistochemical staining to improve recognition of lymphatic vessel invasion. In this study, we used D2-40 immunohistochemical staining to mark breast cancer tissue showing LVI under H&E staining. According to the findings, patients with positive D2-40 staining predicted a higher risk of N-stage and localized recurrence of the tumor. Since positive D2-40 staining suggests the presence of lymphatic vessel invasion, it is reasonable to assume that lymphatic vessel invasion, rather than blood vessel invasion (BVI), is a relevant risk factor for promoting lymph node metastasis and localized recurrence of breast cancer. This offers novel perspectives on how lymphatic vessel invasion affects the progression of breast cancer development. Therefore, we believe that D2-40 could be added to the diagnostic procedures for breast cancer LVI as a complement to H&E staining.

Tumor-associated angiogenesis is one of the mechanisms that contribute to the high degree of invasiveness of breast cancer as it advances. According to Modi et al. [25], there is an important connection between inflammatory breast cancer and the dermal vasculature. They also proposed that the extent of dermal vascular involvement be clarified. Lin et al. [26] concluded that BVI is an independent predictor of poor prognosis in operable breast cancer and is associated with aggressive clinicopathological features. CD34 is a currently recognized sensitive marker of tumor neovascular endothelium, which is present in the endothelial cells of microvessels [27]. In this study, we attempted to utilize CD34 IHC staining to identify LVI in breast cancer. The results of this research, however, did not support the hypothesis that the presence of tumor cells in the bloodstream causes organ metastases in breast cancer. The low sample size of the research and the restriction on the BVI detection rate by CD34 are potential causes of this. Consequently, more research is still required to confirm the correlation between vascular invasion and breast cancer.

Although our study provides novel insights into the predictive significance of LVI in breast cancer, there are limitations that must be taken into account. First off, selection bias could have occurred because all of the cases in this study originated from only one therapy center. Second, even though all study participants received the recommended course of treatments under the guidance and supervision of medical professionals and in accordance with breast cancer management guidelines, we cannot completely rule out the possibility that some patients' failure to comply with the recommended course of treatment due to the side effects of adjuvant therapy may have influenced the study's findings. Finally, the irregular structure of LVI led to a false-negative rate when we used D2-40 and CD34 for specific labeling of LVI in breast cancer. This was because it was impossible to ensure complete labeling of each LVI category during the sampling and staining process. Therefore, deeper investigations may be required to demonstrate the effect of LVI on the prognosis of breast cancer and the significance of specific vascular endothelial markers in research.

## Conclusions

According to this study, LVI is an independent poor prognostic factor in invasive breast cancer and is associated with aggressive clinicopathological characteristics. Furthermore, the metastasis of lymph nodes and the localized recurrence rate of breast cancer are influenced by lymphatic vessel invasion. Immunohistochemical staining for specific vascular endothelial markers can help to classify LVI and advance further research on LVI in breast cancer. According to our findings, LVI is an effective biomarker for predicting the prognosis of breast cancer. Nevertheless, further study is required to clarify and evaluate the mechanisms via which LVI regulates the development and progression of breast cancer.

## Data Availability

All data generated or analyzed in this study are included in the article and further inquiries can be directed to the corresponding author.

## Abbreviations

LVI: Lymphovascular invasion
H&E: Hematoxylin–eosin
IHC: Immunohistochemistry
OS: Overall survival
DFS: Disease-free survival
AJCC: American Joint Committee on Cancer
OR: Odds ratio
HR: Hazard ratio
ER: Estrogen receptor
PR: Progesterone receptor
HER2: Human epidermal growth factor receptor 2
LEC: Lymphatic endothelial cells
BVI: Blood vessel invasion
